# Revision surgery due to failed internal fixation of intertrochanteric femoral fracture: current state-of-the-art

**DOI:** 10.1186/s12891-020-03593-8

**Published:** 2020-08-22

**Authors:** Pei Liu, Dongxu Jin, Changqing Zhang, Youshui Gao

**Affiliations:** Department of Orthopedic Surgery, Shanghai Jiao Tong University Affiliated Sixth People’s Hospital, 600 Yishan Road, Xuhui, Shanghai, 200233 China

**Keywords:** Intertrochanteric femoral fracture, Hip fracture, Revision surgery, Failed fracture fixation, Salvage internal fixation

## Abstract

Failed treatment of intertrochanteric (IT) femoral fractures leads to remarkable disability and pain, and revision surgery is frequently accompanied by higher complication and reoperation rates than primary internal fixation or primary hip arthroplasty. There is an urgent need to establish a profound strategy for the effective surgical management of these fragile patients. Salvage options are determined according to patient physiological age, functional level, life expectancy, nonunion anatomical site, fracture pattern, remaining bone quality, bone stock, and hip joint competency. In physiologically young patients, care should be taken to preserve the vitality of the femoral head with salvage internal fixation; however, for the elderly population, conversion arthroplasty can result in early weight bearing and ambulation and eliminates the risks of delayed fracture healing. Technical challenges include a difficult surgical exposure, removal of broken implants, deformity correction, critical bone defects, poor bone quality, high perioperative fracture risk, and prolonged immobilization. Overall, the salvage of failed internal fixations of IT fractures with properly selected implants and profound techniques can lead to the formulation of valuable surgical strategies and provide patients with satisfactory clinical outcomes.

## Background

Intertrochanteric (IT) fractures are common, accounting for almost half of all hip fractures and resulting in a great burden on orthopedic services [1–3]. Although most IT fractures can be treated successfully with contemporary surgical techniques and internal fixations such as intramedullary nails and sliding hip screws, clinical failures still occasionally occur, with reported data indicating a range from 0.5 to 56% depending on the fracture type, patient status, and quality of the reduction and fixation [4–9]. Failed treatment of IT fractures leads to remarkable disability and pain, which may cause complications associated with prolonged recumbency and affect the vital prognosis of these fragile patients, thereby necessitating effective surgical intervention [10]. Surgery indications include implant failure, nonunion, malunion, fracture, dislocation, femoral head necrosis, posttraumatic arthritis and infection [4, 11, 12]. Salvage osteosynthesis and conversion hip arthroplasty remain the mainstays of treatment for the failed internal fixation of IT fractures rather than conservative, nonoperative therapy, which is limited to incredibly infirm patients [13]. Several technical hurdles emerge in this situation, including residual bone deformity, distorted soft tissue anatomy, broken implants, poor bone stock, and femoral deficiency. Accordingly, management of these cases has been reported with increased risks of perioperative morbidity, prolonged operative times, escalated blood loss, frequent intraoperative fracture, and a high rate of early dislocation [14, 15]. In this review, we discuss novel strategies regarding salvage options and surgical techniques to improve the outcome of patients with failed internal fixations of IT fractures.

## Salvage options

**Fig. 1:**
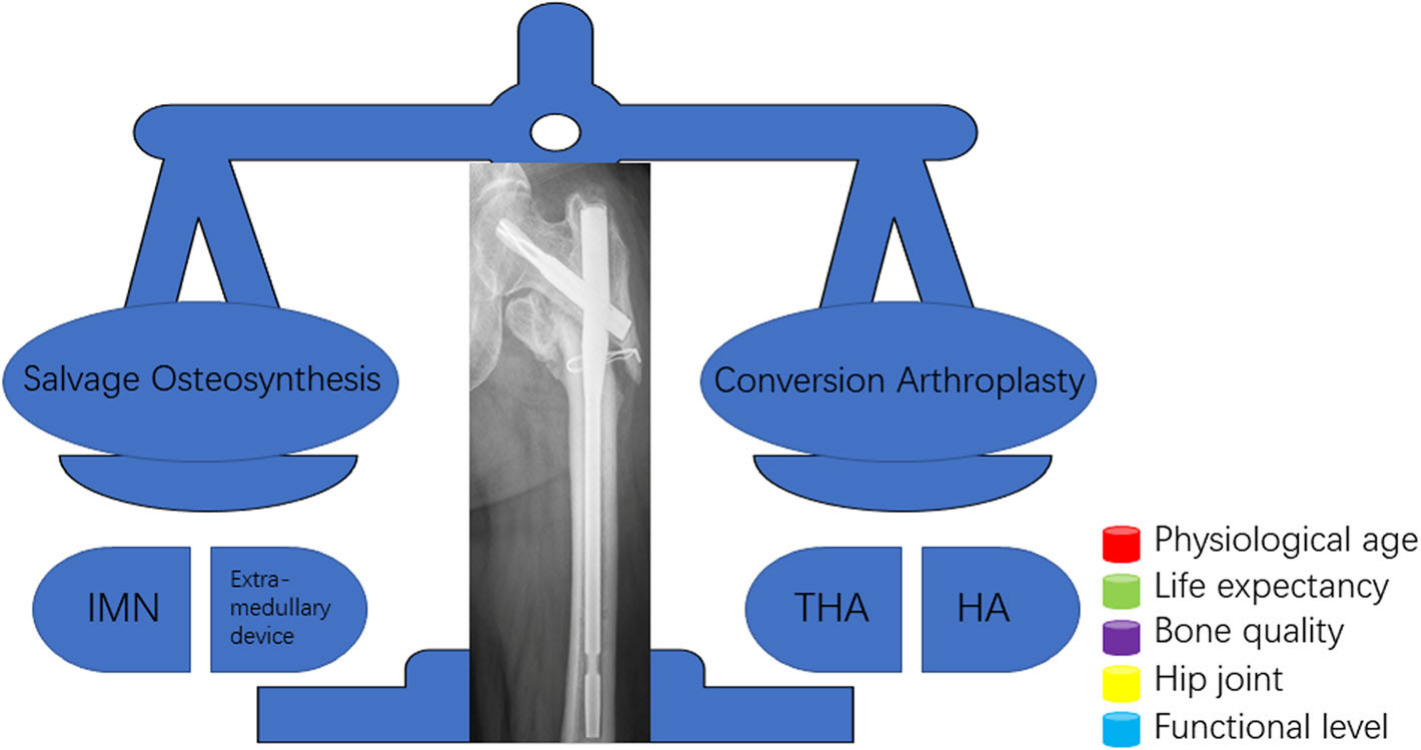
The strategy to treat failed osteosynthesis of intertrochanteric fractures is weighed between salvage osteosynthesis and conversion arthroplasty. The decision is multifactorial and should be individualized

In properly selected patients, a high rate of successful revision surgery can be achieved [1, 5, 16]. The decision to perform either revision osteosynthesis or prosthetic replacement is based on multiple factors: patient physiological age, functional level, life expectancy, nonunion anatomical site, fracture pattern, remaining bone quality, bone stock, and hip joint competency (Fig. 1) [17, 18]. Briefly, salvage osteosynthesis is preferable for physiologically young patients with long life expectancies and adequate bone quality for fixation; hip arthroplasty, in contrast, is preferred for the geriatric population with poor bone quality, inadequate bone stock, and severely damaged hip articular surfaces. Conversion hip arthroplasty is beneficial for early weight bearing and mobilization, eliminating the risks of delayed fracture healing and accelerating functional recovery, which are pivotal for prognosis in elderly, debilitated patients [1, 2, 10].

### Femoral head salvage procedures

Failure of fixation of IT fractures in young patients is exceedingly rare [19, 20]. However, open reduction and revision internal fixation with or without osteotomy or bone grafting have been reported to achieve high union rates and few complications [5]. Unlike hip arthroplasty, which is characterized by limited longevity, revision internal fixation preserves the femoral head; thus, further revision surgery due to prosthesis abrasion is unnecessary. In this regard, orthopedic surgeons should spare no effort to preserve native bone and achieve fracture union with this procedure, especially in active patients younger than 50 years of age [2, 21].

Implants used for revision internal fixation are typically selected according to the quality and location of the remaining bone stock of the proximal femur. The bone stock of the inferior femoral head has usually not been violated by the prior device. Fixed angle devices, such as angled blade plates and dynamic condylar screws (DCSs), are preferred and often accompanied by autogenous bone grafting [17]. Multiple cervicodiaphyseal angles are available for fixation of the proximal bone fragment according to the preoperative plan. Alternatively, it is possible to reinsert sliding hip screws, such as dynamic hip screws (DHSs), if there is adequate bone stock in the femoral head to hold another screw [22]. In one study, 26 patients with failed DHS fixations of IT fractures were included [22]. Eighteen patients were treated with revision internal fixations, and 8 patients were treated with prosthetic replacements. Among the revision internal fixation group, DHS reinsertion was used in 8 patients, valgus osteotomy and revision DHS fixation in 6 patients, and valgus osteotomy and insertion of a single-angled 130° plate in 4 patients. All patients in the revision internal fixation group achieved fracture union without bone grafting at a mean time of 17 weeks. Four of 18 patients had occasional hip pain that did not interfere with their daily activities, and the rest were pain-free after a femoral head salvage procedure at the last follow-up. All 18 patients could walk without support at the final follow-up. In another series of 20 patients with failed IT fractures, repeat open reductions and internal fixations (angled blade plates in 11 patients, DHSs in 5, DCSs in 3 and a Zickel nail in 1) with bone grafting were evaluated [5]. Nineteen of 20 nonunions healed, and 16 of 19 patients who achieved fracture union reported no pain, while the other 3 had mild pain (related to the retained implant). All were ambulatory. In a recent retrospective study, 11 salvages for nail breakage were identified [23]. Salvage procedures included conservative treatment in 2 cases, an intramedullary long nail in 4, 95° DCSs in 3 and conversion total hip arthroplasty in 2. All revision internal fixation devices were combined with decortication and bone grafting. The main Barthel score improved from 63.2 preoperatively to 72.8 postoperatively. Regarding the SF-12 score collected at the final follow-up, the physical summation was 36.43, and the mental summation was 35.83. A better result in the Bodily Pain (0.708, *p* = 0.049) and Role-Emotional (0.815, *p* = 0.01) subscores in the SF-12 score was observed among the population with an elevated Barthel score. All of the above literature demonstrates that fracture union and a good outcome can be achieved with revision internal fixation for physiologically young patients and even some older patients with good remaining bone stock.

Intramedullary nails have a role in revision surgery. They are characterized by a short lever arm, with as much as a 30% reduction in bending stresses with respect to that of extramedullary devices. Additionally, they act as an intramedullary buttress to avoid excessive shaft medialization. Some advocates claim they have clinical benefits such as minimal surgical exposure, prevention of fracture hematoma, less blood loss, lower pain scores, improved functional ability and early mobilization [24, 25]. Most recently, 20 failed intramedullary nail fixations were examined through 4 different revision procedures, including proximal femoral locking plates in 6 patients, intramedullary nails in 8 (40%) and prosthesis replacement in 6 [24]. According to the radiographic follow-up, fracture union in the repeated nailing group was observed at a mean period of 118.6 days, while the plate revision group required a longer time of 427.6 days. Barthel scores decreased from the third month to the twelfth month postoperatively; however, the result was not statistically significant. Importantly, the mortality rates of the nail group (25%) were lower than those of the plate group (33%) and arthroplasty group (33%) 12 months after revision surgery. This study illustrates that intramedullary nails may have a slight advantage in terms of lower mortality and could therefore be a beneficial option when treating failed nail fixation in these frail patients.

**Fig. 2:**
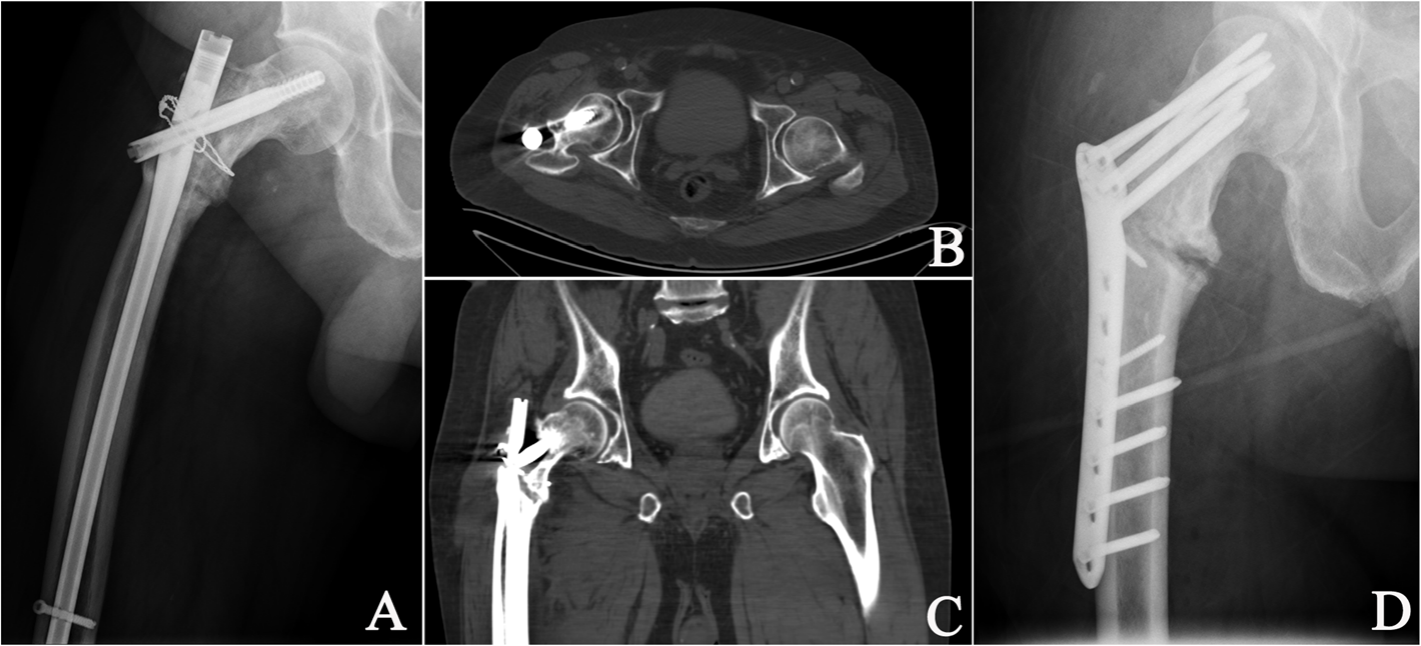
Failed nailing of an intertrochanteric fracture in an active 78-year-old man. **a** The intertrochanteric fracture had been stabilized by an antegrade long γ nail and circumferential cerclage 4 years ago. The patient first experienced significant hip pain and restricted hip motion 1 month ago without trauma history. Radiography showed nonunion of the intertrochanteric fracture and breakage of the γ nail. **b** Transverse and **c** coronal sections of CT scans showed osteolysis of the great trochanter as well as coxa vara with obvious fracture gaps. **d** Prior implants were removed. The malalignment was corrected, causing a larger gap in the calcar. Sufficient bone grafting was used to fill the gap, and the fracture was stabilized by a reverse LISS for the distal femur

Locking plate systems are useful alternatives for revision internal fixation of IT fractures. Although the biomechanical superiority of the intramedullary nail is substantial, locking plates provide sufficient stability to maintain the alignment of the proximal femur, with a low demand for the entrance point and medullary canal (Fig. 2). In our own experience, the time to full weight bearing should be postponed when radiographic callus formation is distinguishable. However, active functional exercises can be initiated immediately after the operation. All beneficial maneuvers to promote fracture healing can be attempted postoperatively.

Some authors have proposed additional cement augmentation around the blade tip to enhance anchorage in the remaining bone of the femoral head in specified cases, including lateral blade migration or peri-implant fracture [26, 27]. Rotational stability and pull-out strength increased after augmentation of the previously extracted proximal femur nail antirotation (PFNA) blade based on biomechanical investigations [26]. In a study enrolling 10 patients with failed proximal femoral fractures, revision surgeries were conducted via augmented PFNA [27]. Fracture healing was documented in all cases except for 2 patients who died from heart failure before full consolidation during a limited follow-up period of 5.4 months. No negative biological side effects were observed from the cement. In certain cases, proceeding revision surgery with either blade exchange or cement augmentation should be prudent. Salvage options including blade exchange, cement-augmented PFNA, PFNA renailing and arthroplasty were investigated in 57 cases with blade ‘cut-out’ and ‘cut-through’ after fixation of IT fractures with PFNA or trochanter fixation nails (TFNs) [28]. In the ‘cut-through’ group, 8 of 16 patients (50%) who were revised with blade exchange and 2 of 6 (33%) revised with blade exchange and augmentation required further revision operations. Total hip arthroplasty (THA) was the definitive treatment in all cases. In the ‘cut-out’ group, 2 of 3 patients (66%) revised with blade exchange and 2 of 3 (66%) who underwent renailing required additional procedures. Resistance to axial migration of the blade is minimal when the blade has already perforated through the femoral head cortex (cut-through). The residual bone stock may not have sufficient strength to hold another blade; thus, high reoperation rates are inevitable after blade exchange. Collectively, the data on revision intramedullary nails are either scarce or include too few cases for broad interpretability of the results. Further multicentric studies with prospective designs may offer improved treatment for patients with failed IT fractures.

### Conversion hip arthroplasty

Clinical failure of internal fixation of IT fractures is relatively common in osteopenic elderly patients. As the success of revision osteosynthesis is limited by the host’s healing capacity, salvage arthroplasty becomes a reasonable treatment alternative in this affected population [14, 29, 30].

**Fig. 3:**
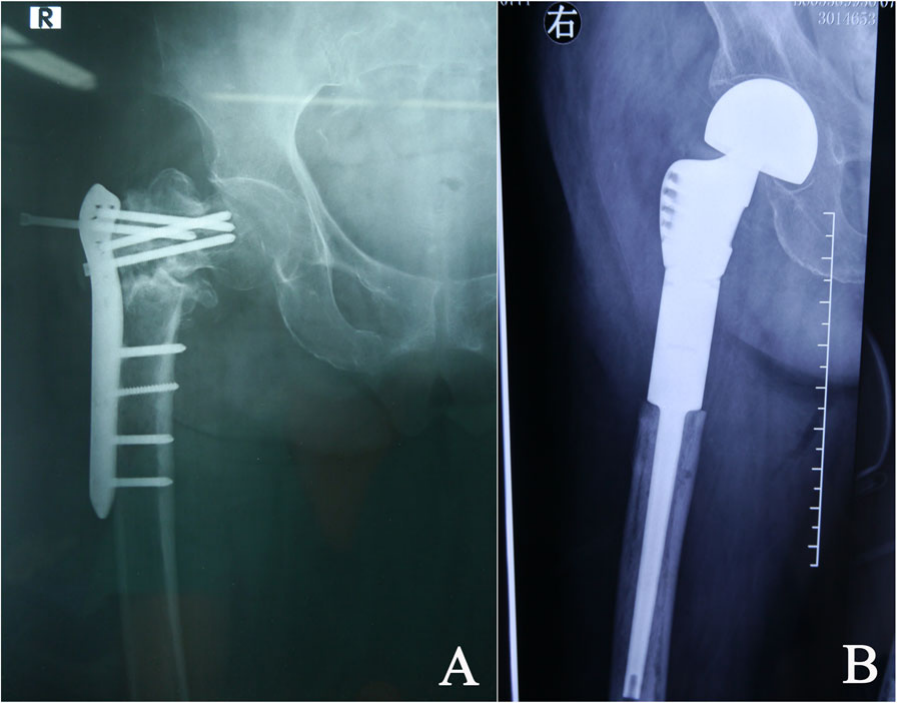
Failed plate osteosynthesis of an intertrochanteric fracture in a 77-year-old lady. **a** Obvious sclerosis, coxa vara, limb shortening, and screw pull-out was observed, indicating nonunion of the intertrochanteric fracture. Significant osteoporosis should be noted, as reflected by the thin femoral cortices. The patient’s painful limping was evident. **b** A cemented modular long-stem prosthesis was used to restore the limb length and facilitate enhanced recovery after the surgery

The decision to perform a hemiarthroplasty (HA) or a THA should be made based on the functional demand of the patient and the status of the acetabular articular cartilage (Fig. 3). With well-preserved cartilage, HA may be considered, providing minimal invasiveness and desirable stability in patients with several comorbidities and low activity demand [17]. A previous study reported that 16 patients with failed hip screw fixation of IT fractures were treated by HA [31]. HA was selected since the femoral head was evacuated by the loosened lag screw. However, the acetabular cartilage was found to be intact during the surgery. All patients experienced functional improvement based on the SF-36 questionnaire score, which increased from 41.9 to 82.7. In cases of badly damaged articular cartilage of the hip, such as preexisting degenerative arthritis and erosion caused by metalwork penetration, THA was the better choice. As mentioned above, THA was recommended as the ideal salvage procedure for ‘cut-out’ and ‘cut-through’ of helical blades after fixation of IT fractures with PFNA and TFN over revision nail fixation, which often has an unacceptable reoperation rate [28]. Recently, a meta-analysis was performed to compare the outcome between THA and HA for failed internal fixations of IT fractures [14]. Six studies with 188 patients (100 THA and 88 HA) were analyzed. No significant difference was found between THA and HA based on postoperative dislocations, reoperations, infections, intraoperative or postoperative fractures, and stem subsidence. Harris Hip Scores were slightly higher in THA than in HA at a minimum 14-month follow-up. This study elucidated that both THA and HA are effective salvage procedures for these specific populations.

Successful femoral component fixation can be achieved with either cemented or cementless implants [21, 30, 32]. In a series of 33 conversion arthroplasties, cementless prostheses were used due to a concern for cement-related cardiopulmonary complications [6]. At the time of the last follow-up, all acetabular cups and femoral stems demonstrated radiographic bone ingrown stability. No detectable wear or periprosthetic osteolysis was observed, and none of the patients underwent component dislocations. Cement fixation is more suitable for relatively elderly patients (generally more than 70 years of age), especially when bone quality is poor and the canal is capacious [11, 17].

Various prostheses with different special designs have been reported, and most of them can yield ideal outcomes with few complications. Surgeons may determine the most suitable prosthesis according to its advantages and disadvantages when facing unique clinical cases. Modular implants enable separate preparation of the proximal and distal bone in the femur to maximize prosthesis filling. Additionally, modular stems may be individually adjusted for leg length discrepancies (LLDs), offsets, anteversions, and proximal femoral bone loss [33, 34]. Cementless modular stems designed for metaphysodiaphyseal anchorage were investigated in the salvage of 29 patients after failed internal fixations of IT fractures [35]. During a mean follow-up of 20 months, all the patients reported notable pain relief and functional improvement, indicating that the modular stem was a reliable implant. Furthermore, another study involving 11 patients confirmed modular arthroplasty as an effective salvage procedure [1].

Nonmodular revision prostheses are also a rational option. Since they are simple to assemble during surgery, there is no concern for fracture of the modular stem at the mid-stem junction. In a retrospective study of 31 failed IT fixations, all patients were salvaged using nonmodular cementless long-stem distal fixation [36]. After a mean follow-up of 47.5 months, all patients reported significant pain relief and a return to ambulation. The Harris Hip score increased from 28.4 to 85.6 postoperatively. Radiological records showed that all presented with bony union.

### Technical challenges and considerations

Revision surgery for failed internal fixation of IT fractures is a challenging and highly demanding procedure. Orthopedic surgeons often face technical hurdles, including the removal of broken fixation devices, a difficult surgical exposure, altered anatomy, compromised bone quality due to pre-existing osteopenia, bone defects after the extraction of failed implants, new device placement, a high perioperative fracture risk, and prolonged immobilization. Attention to technical details can minimize potential complications [17].

The initial exposure is complicated by the presence of prior fixed metalwork and anatomical deformities [37]. The status of the greater trochanter is important: it can be malunited and block the intramedullary canal or completely ununited. If trochanter malunion prevents adequate preparation of the proximal femur, a trochanteric sliding osteotomy can be useful [19, 38]. This technique preserves the continuity of the abductors, the trochanter and the vastus lateralis, which are important in maintaining hip stability. As a result, the possibility of postoperative hip dislocation is reduced. Retraction of the overhanging trochanter, as an obstacle during femoral preparation, can provide excellent exposure of the hip joint. In a study involving 71 failed IT fracture treatments, trochanteric sliding osteotomy was performed in 22% of patients during salvage surgery, aiming to facilitate exposure of the hip. None of the patients experienced greater trochanteric fractures or neurovascular injuries after surgery [39].

When performing IT salvage procedures, the removal of failed fixation devices involves a more extensive dissection and frequently requires the removal of broken screws. It is helpful to prepare instruments such as trephines, grasping tools, standard broken screw removal sets, and metal-cutting high-speed burrs ahead of time [37, 40]. Several studies recommended dislocating the femoral head before extracting the implant, which may reduce the possibility of intraoperative fracture if conversion arthroplasty is planned [6]. A technical report provided more details on lag screw removal for failed DHS revisions [41]. Briefly, after removing the DHS plate and carefully dissecting the soft tissue with subsequent hip dislocation, saw cuts are made around the lag screw in 4 different directions (superior, anterior, inferior and posterior) of the femoral neck. Next, the femoral head is simply removed by straight traction with the screw in situ. This avoids large torques when a traditional backing screw out is applied.

If the decision is made to proceed with revision internal fixation, it is important to obtain stable fixation of the fracture fragments and avoid varus malreduction [19]. Eliminating the fracture gap by means of a compression technique and sufficient bone grafting and taking care to preserve the vascularity of fracture sites could jointly improve the environment for fracture healing [2, 42].

For conversion arthroplasty, there are several pitfalls to consider when preparing the femoral canal. Fracture callus, nonunited fracture translation, and malunion often result in bone deformity of the proximal femur, which increases the risk of intraoperative fracture during canal preparation or implant placement [39, 43]. Thus, trochanteric fragments and distorted anatomies must be mobilized before opening the femoral canal. Careful dissection aiming to avoid damaging adjacent neurovascular structures and muscles is required during this process [39]. It might be difficult to estimate the correct relationship between comminuted fracture patterns for reconstructing the proximal femoral anatomy; however, restoring the relationship of the tip of the greater trochanter and the center of rotation of the femoral head can indicate a reasonable reference point. Elaborate intraoperative trials and imaging are encouraged to produce a successful surgery [19]. Endosteal sclerotic bone along the track of the previous intramedullary nails and lag screws may lead to fracture of the femur and/or displacement of the stem during its insertion [6]. A gauge osteotome and/or burr is useful to remove the endosteal bone. The horizontal axis of the knee joint can be used as the reference rather than the deformed axis of the femoral neck when adjusting the anteversion of the stem [6]. The femoral canal can be opened using a high-speed burr and hand reamed with the reamer length selected according to the preoperative templating to obtain an optimal endosteal contact in the distal diaphyseal part of the femur. A C-arm image intensifier or fluoroscopic image intensifier is helpful in guiding this process, as the index surgery can result in an abnormal proximal femoral anatomy, medullary canal obstruction and stress-riser formation [10, 30].

Bone defects of the proximal femur after implant removal are challenging events in revision surgeries of failed IT fixations (Fig. 2). Either intramedullary bone defects or cortical screw holes should be taken into consideration to obtain a successful surgery. Bone loss distal to the standard neck resection level often requires revision-type implants, including calcar-replacing implants, to make up for any bone deficiency and restore limb length. Long-stem implants are inserted to bypass the most distal screw hole by two cortical diameters, combined with or without a prophylactic cable to avoid creation of a stress riser [17, 44]. In a series of 71 affected hips treated with conversion THA, 76% calcar-replacing prostheses and 50% long-stem components were reported [39]. Similarly, 14/21 long-stem implants were claimed in a study reported by D’Arrigo and colleagues [45]. Likewise, calcar replacement was used in almost 60% of patients treated with salvage arthroplasty for failed IT fixations and long-stem implants in 50% according to an investigation enrolling 61 cases [43]. Taken together, calcar-replacing implants and long-stem designs have been widely applied for revision arthroplasty in patients with failed internal fixations of IT fractures. Moreover, tumor-specific endoprostheses are an alternative option for managing patients with inadequate proximal femoral bone stock. They has been shown to be of significant benefit with a mean Oxford Hip Score of 33 for patients with failed osteosynthesis of proximal femoral fractures in a 5-year follow-up study [46].

Researchers have drawn attention to the fact that cement extrudes from empty screw holes when a cemented stem is used. Leakage of cement through screw holes may lead to nonunion at the fracture site, postoperative periprosthetic fracture or loss of cement pressurization [6]. Numerous techniques for preventing this extrusion have been advocated: for screw holes, direct finger pressure, gauze, and reinsertion of the screws can be used to plug the holes when cement is injected; for lag screw holes, the assistant’s thumb, firmly packed gauze, a surgical glove inflated with saline, and a bone plug fashioned from the excised femoral head are valid [47, 48].

Acetabular bone quality in patients with IT nonunion is also compromised because of disuse osteopenia. If a cementless cup is used, inadequate press-fit fixation or intraoperative fracture during implant fixation can occur. Reaming acetabular cartilage judiciously and aiming to preserve the subchondral bone are recommended. Forceful acetabular component impaction is not allowed; instead, augmentation of the fixation with screws should be considered [17, 37].

A separated greater trochanter, commonly seen in patients subjected to IT fixation failure, usually causes pain and limping and even affects abductor function [31, 49]. Additionally, evidence has revealed that a higher dislocation rate of hip arthroplasty is correlated with displaced fracture of the greater trochanter [31]. Three methods for fixation of the greater trochanter have been mentioned: contoured plating, tension band wiring, and trochanter claw plating with wiring [11, 30, 31, 35, 36, 46]. In a retrospective study of 16 failed IT fixations, all patients received surgery with HA and the cable-grip system. Fifteen out of 16 patients were observed to have solid union of the greater trochanter postoperatively by 24 weeks, and no dislocation of HA occurred during the follow-up [31].

A summary of technical challenges and corresponding strategies is presented in Table 1.

**Table 1:** Technical challenges and strategies

Challenge	Strategy	Reference
Surgical exposure	Trochanteric sliding osteotomy	[19, 38]
Removal of previous fixation devices	Dislocating the hip joint before removing; excising the femoral head with the lag screw in situ	[6, 41]
Removal of broken screws	Trephines, grasping tools, a standard broken screw removal set and a metal-cutting high-speed burr	[37, 40]
Revision internal fixation	Avoiding a varus malreduction and obtaining stable fixation (compression technique and bone grafting)	[2, 19, 42]
Bone deformity of proximal femur	Restoring the relationship between the tip of greater trochanter and the center of femoral head rotation	[19]
Femoral canal preparation for revision arthroplasty	Endosteal sclerotic bone removal: gauge osteotome and/or a burr; refereing horizontal axis of the knee joint to adjust the anteversion of the stem; using C-arm image intensifier or fluoroscopic image intensifier to guide the placement of the stem	[6, 10, 30]
Bone defect of proximal femur	Calcar-replacing and long-stem implant combined with or without a prophylactic cable; tumor-type endoprosthesis	[17, 39, 44, 46]
Leakage of cement through screw holes	Finger pressure, packed gauze, re-inserted screws, surgical glove inflated with saline, fashioned bone plug	[47, 48]
Acetabula preparation in patients with poor bone quality	Reaming acetabular cartilage judiciously; avoiding forceful component impaction; considering screws augmentation	[17, 37]
Greater trochanter reattachment	Contoured plating, tension band wiring and trochanter claw plate with wiring	[11, 30, 35, 36, 46]

## Conclusion

Although failed internal fixations of IT fractures are rarely reported, the relatively higher complication rates, reoperation rates and surgical hurdles collectively make them challenging for both orthopedic surgeons and affected patients. The recent publication of investigations has provided valuable strategies based on salvage options and surgical techniques. In physiologically young patients, efforts should be made to preserve the femoral head with salvage internal fixation; however, for the geriatric population, conversion arthroplasty offers the opportunity for early weight bearing and ambulation, which are of paramount importance for the improvement of both morbidity and mortality. Additionally, attention to specific techniques is important for establishing a considerate, effective salvage strategy.

## Data Availability

The data and materials supporting the conclusions of this article are included within the article.

## Abbreviations

DCS: Dynamic condylar screw
DHS: Dynamic hip screw
HA: Hemiarthroplasty
IT: Intertrochanteric
LLD: Leg length discrepancy
PFNA: Proximal femur nail antirotation
TFN: Trochanter fixation nail
THA: Total hip arthroplasty

## References

[1] Karampinas PK, Kollias G, Vlamis J, Papadelis EA, Pneumaticos SG (2015). Salvage of failed hip osteosynthesis for fractures with modular hip prosthesis. Eur J Orthop Surg Traumatol.

[2] Dziadosz D (2015). Considerations with failed intertrochanteric and subtrochanteric femur fractures: how to treat, revise, and replace. J Orthop Trauma.

[3] Leer-Salvesen S, Engesaeter LB, Dybvik E, Furnes O, Kristensen TB, Gjertsen JE (2019). Does time from fracture to surgery affect mortality and intraoperative medical complications for hip fracture patients? An observational study of 73 557 patients reported to the Norwegian Hip Fracture Register. Bone Joint J.

[4] Bercik MJ, Miller AG, Muffly M, Parvizi J, Orozco F, Ong A (2012). Conversion total hip arthroplasty: a reason not to use cephalomedullary nails. J Arthroplast.

[5] Haidukewych GJ, Berry DJ (2003). Salvage of failed internal fixation of intertrochanteric hip fractures. Clin Orthop Relat Res.

[6] Lee YK, Kim JT, Alkitaini AA, Kim KC, Ha YC, Koo KH (2017). Conversion hip arthroplasty in failed fixation of intertrochanteric fracture: a propensity score matching study. J Arthroplast.

[7] Kiriakopoulos E, McCormick F, Nwachukwu BU, Erickson BJ, Caravella J (2017). In-hospital mortality risk of intertrochanteric hip fractures: a comprehensive review of the US Medicare database from 2005 to 2010. Musculoskelet Surg.

[8] Lin JC, Liang WM (2017). Mortality, readmission, and reoperation after hip fracture in nonagenarians. BMC Musculoskelet Disord.

[9] Puram C, Pradhan C, Patil A, Sodhai V, Sancheti P, Shyam A (2017). Outcomes of dynamic hip screw augmented with trochanteric wiring for treatment of unstable type A2 intertrochanteric femur fractures. Injury.

[10] Thakur RR, Deshmukh AJ, Goyal A, Ranawat AS, Rasquinha VJ, Rodriguez JA (2011). Management of failed trochanteric fracture fixation with cementless modular hip arthroplasty using a distally fixing stem. J Arthroplast.

[11] Muller F, Galler M, Zellner M, Bauml C, Fuchtmeier B (2017). Total hip arthroplasty after failed osteosynthesis of proximal femoral fractures: revision and mortality of 80 patients. J Orthop Surg.

[12] Morice A, Ducellier F, Bizot P, Orthopaedics, Traumatology Society of Western F (2018). Total hip arthroplasty after failed fixation of a proximal femur fracture: analysis of 59 cases of intra- and extra-capsular fractures. Orthop Traumatol Surg Res.

[13] Smith A, Denehy K, Ong KL, Lau E, Hagan D, Malkani A (2019). Total hip arthroplasty following failed intertrochanteric hip fracture fixation treated with a cephalomedullary nail. Bone Joint J.

[14] Luthringer TA, Elbuluk AM, Behery OA, Cizmic Z, Deshmukh AJ (2018). Salvage of failed internal fixation of intertrochanteric hip fractures: clinical and functional outcomes of total hip arthroplasty versus hemiarthroplasty. Arthroplasty Today.

[15] Xu Q, Lai J, Zhang F, Xu Y, Zhu F, Lin J, Zhao M, Ye J, Wen L. Poor outcomes for osteoporotic patients undergoing conversion total hip arthroplasty following prior failed dynamic hip screw fixation: a nationwide retrospective cohort study. J Int Med Res. 2019:300060518823410. 10.1177/0300060518823410. 10.1177/0300060518823410 PMC6460592 30669904

[16] Bhowmick K, Matthai T, Boopalan PRJ, Jepegnanam TS. Decision making in the management of malunion and nonunion of intertrochanteric fractures of the hip. Hip Int. 2019:1120700019863410. 10.1177/1120700019863410. 10.1177/1120700019863410 31304795

[17] Haidukewych GJ, Berry DJ (2005). Salvage of failed treatment of hip fractures. J Am Acad Orthop Surg.

[18] Iwakura T, Niikura T, Lee SY, Sakai Y, Nishida K, Kuroda R, Kurosaka M (2013). Breakage of a third generation gamma nail: a case report and review of the literature. Case Rep Orthop.

[19] Petrie J, Sassoon A, Haidukewych GJ (2013). When femoral fracture fixation fails: salvage options. Bone Joint J.

[20] Park JS, Lee HS, Won SH, Lee DW, Jung KJ, Kim CH, Kim JH, Lee WS, Ryu A, Kim WJ (2019). Intertrochanteric fracture with low-energy trauma in a young woman with anorexia nervosa: a case report. Medicine.

[21] Angelini M, McKee MD, Waddell JP, Haidukewych G, Schemitsch EH (2009). Salvage of failed hip fracture fixation. J Orthop Trauma.

[22] Said GZ, Farouk O, El-Sayed A, Said HG (2006). Salvage of failed dynamic hip screw fixation of intertrochanteric fractures. Injury.

[23] Cruz-Sanchez M, Torres-Claramunt R, Alier-Fabrego A, Martinez-Diaz S (2015). Salvage for nail breakage in femoral intramedullary nailing. Injury.

[24] Tucker A, Warnock M, McDonald S, Cusick L, Foster AP (2018). Fatigue failure of the cephalomedullary nail: revision options, outcomes and review of the literature. Eur J Orthop Surg Traumatol.

[25] Yu X, Wang H, Duan X, Liu M, Xiang Z (2018). Intramedullary versus extramedullary internal fixation for unstable intertrochanteric fracture, a meta-analysis. Acta Orthop Traumatol Turc.

[26] Erhart S, Kammerlander C, El-Attal R, Schmoelz W (2012). Is augmentation a possible salvage procedure after lateral migration of the proximal femur nail antirotation?. Arch Orthop Trauma Surg.

[27] Scola A, Gebhard F, Dehner C, Roderer G (2014). The PFNA(R) augmented in revision surgery of proximal femur fractures. Open Orthop J.

[28] Brunner A, Buttler M, Lehmann U, Frei HC, Kratter R, Di Lazzaro M, Scola A, Sermon A, Attal R (2016). What is the optimal salvage procedure for cut-out after surgical fixation of trochanteric fractures with the PFNA or TFN?: a multicentre study. Injury.

[29] Sayac G, Neri T, Schneider L, Philippot R, Farizon F, Boyer B. Low revision rates at more than 10 years for dual-mobility cups cemented into cages in complex revision total hip arthroplasty. J Arthroplast. 2019. 10.1016/j.arth.2019.08.058. 10.1016/j.arth.2019.08.058 31543421

[30] Moon NH, Shin WC, Kim JS, Woo SH, Son SM, Suh KT (2019). Cementless total hip arthroplasty following failed internal fixation for femoral neck and intertrochanteric fractures: a comparative study with 3-13 years’ follow-up of 96 consecutive patients. Injury.

[31] Hsu CJ, Chou WY, Chiou CP, Chang WN, Wong CY (2008). Hemi-arthroplasty with supplemental fixation of greater trochanter to treat failed hip screws of femoral intertrochanteric fracture. Arch Orthop Trauma Surg.

[32] Zeng X, Zhan K, Zhang L, Zeng D, Yu W, Zhang X, Zhao M (2017). Conversion to total hip arthroplasty after failed proximal femoral nail antirotations or dynamic hip screw fixations for stable intertrochanteric femur fractures: a retrospective study with a minimum follow-up of 3 years. BMC Musculoskelet Disord.

[33] Weiss RJ, Karrholm J, Hailer NP, Beckman MO, Stark A (2012). Salvage of failed trochanteric and subtrochanteric fractures using a distally fixed, modular, uncemented hip revision stem. Acta Orthop.

[34] Tetsunaga T, Fujiwara K, Endo H, Noda T, Tetsunaga T, Sato T, Shiota N, Ozaki T (2017). Total hip arthroplasty after failed treatment of proximal femur fracture. Arch Orthop Trauma Surg.

[35] Laffosse JM, Molinier F, Tricoire JL, Bonnevialle N, Chiron P, Puget J (2007). Cementless modular hip arthroplasty as a salvage operation for failed internal fixation of trochanteric fractures in elderly patients. Acta Orthop Belg.

[36] Shi X, Zhou Z, Yang J, Shen B, Kang P, Pei F (2015). Total hip arthroplasty using non-modular cementless long-stem distal fixation for salvage of failed internal fixation of intertrochanteric fracture. J Arthroplast.

[37] Krause PC, Braud JL, Whatley JM (2015). Total hip arthroplasty after previous fracture surgery. Orthop Clin North Am.

[38] Lakstein D, Backstein DJ, Safir O, Kosashvili Y, Gross AE (2010). Modified trochanteric slide for complex hip arthroplasty: clinical outcomes and complication rates. J Arthroplast.

[39] Mortazavi SM, Bican O, Kane P, Parvizi J, Hozack WJ, M RG (2012). Total hip arthroplasty after prior surgical treatment of hip fracture is it always challenging?. J Arthroplast.

[40] Yuan BJ, Abdel MP, Cross WW, Berry DJ (2017). Hip arthroplasty after surgical treatment of intertrochanteric hip fractures. J Arthroplast.

[41] Campbell AC, Goyal S, Miller NJ, Sinha S (2010). New technique for revising dynamic hip screw fixations with lag screw in situ. J Orthop Trauma.

[42] Xue D, Yu J, Zheng Q, Feng G, Li W, Pan Z, Wang J, Li H (2017). The treatment strategies of intertrochanteric fractures nonunion: an experience of 23 nonunion patients. Injury.

[43] Haidukewych GJ, Berry DJ (2003). Hip arthroplasty for salvage of failed treatment of intertrochanteric hip fractures. J Bone Joint Surg Am.

[44] Mingli F, Huiliang S, Guanglei C, Zheng L, Shibao L, Limin L, Shuai A (2017). A clinical study on arthroplasty for failed internal fixation of hip fractures and review of literature. Pak J Med Sci.

[45] D'Arrigo C, Perugia D, Carcangiu A, Monaco E, Speranza A, Ferretti A (2010). Hip arthroplasty for failed treatment of proximal femoral fractures. Int Orthop.

[46] Grammatopoulos G, Alvand A, Martin H, Whitwell D, Taylor A, Gibbons CL (2016). Five-year outcome of proximal femoral endoprosthetic arthroplasty for non-tumour indications. Bone Joint J.

[47] Zhang B, Chiu KY, Wang M (2004). Hip arthroplasty for failed internal fixation of intertrochanteric fractures. J Arthroplast.

[48] Langdown AJ, Low AK, Auld JW, Bruce WJ, Walker PM (2005). Technique for preventing cement extrusion from screw holes during conversion of failed hip fracture fixation to total hip replacement. Ann R Coll Surg Engl.

[49] Ren H, Huang Q, He J, Wang Y, Wu L, Yu B, Zhang D (2019). Does isolated greater trochanter implication affect hip abducent strength and functions in intertrochanteric fracture?. BMC Musculoskelet Disord.

